# Integrating Care in Norfolk (ICN)—A case study on a two-year programme to improve integrated working between primary care, community health services and social care in six localities

**Published:** 2012-09-04

**Authors:** Helen Tucker, Mark Burgis

**Affiliations:** Project Consultant “Integrating Care in Norfolk”, Norfolk County Council and NHS Norfolk; Researcher University of Warwick, UK; Senior Project Manager “Integrating Care in Norfolk”, Norfolk County Council and NHS Norfolk—currently Chief Executive Officer Mid Norfolk Consortium, UK

**Keywords:** primary care, social care, community services, integrated hub

## Abstract

The ICN programme was designed to test ways of integrating health and social care services in six localities with a population of nearly 300,000 residents with the objective of improving patient and staff satisfaction, and reducing demands on secondary care. The ICN programme is one of 16 national integrated care pilots in England.

Data were sourced from questionnaires, focus groups, interviews, and recorded service activity. A core group of practitioners in each locality focused on redesigning services according to their local circumstances in order to improve care for adults and older people with complex care needs. Local arrangements included developing multidisciplinary teams based in GP practices, with coordinators operating in a local hub. 845 patients received ICN interventions.

The study showed high levels of satisfaction from patients and staff, with surveyed staff being unanimous that this way of working should continue. A study of 12 of the 32 GP practices that engaged early in the programme showed a 31% reduction in unplanned admissions to hospital between the first and second year of the pilot. Issues included the time required for building trust and communication, the importance of building a platform for integrating care locally and extending to a whole-system approach.

Powerpoint presentation available at http://www.integratedcare.org at congresses – San Marino – programme.

